# A Novel Intervention Including Individualized Nutritional Recommendations Reduces Hemoglobin A1c Level, Medication Use, and Weight in Type 2 Diabetes

**DOI:** 10.2196/diabetes.6981

**Published:** 2017-03-07

**Authors:** Amy L McKenzie, Sarah J Hallberg, Brent C Creighton, Brittanie M Volk, Theresa M Link, Marcy K Abner, Roberta M Glon, James P McCarter, Jeff S Volek, Stephen D Phinney

**Affiliations:** 1 Virta Health San Francisco, CA United States; 2 Indiana University Health Arnett Medically Supervised Weight Loss Lafayette, IN United States

**Keywords:** type 2 diabetes, ketosis, Hb A1c, weight loss, mobile health

## Abstract

**Background:**

Type 2 diabetes (T2D) is typically managed with a reduced fat diet plus glucose-lowering medications, the latter often promoting weight gain.

**Objective:**

We evaluated whether individuals with T2D could be taught by either on-site group or remote means to sustain adequate carbohydrate restriction to achieve nutritional ketosis as part of a comprehensive intervention, thereby improving glycemic control, decreasing medication use, and allowing clinically relevant weight loss.

**Methods:**

This study was a nonrandomized, parallel arm, outpatient intervention. Adults with T2D (N=262; mean age 54, SD 8, years; mean body mass index 41, SD 8, kg·m^−2^; 66.8% (175/262) women) were enrolled in an outpatient protocol providing intensive nutrition and behavioral counseling, digital coaching and education platform, and physician-guided medication management. A total of 238 participants completed the first 10 weeks. Body weight, capillary blood glucose, and beta-hydroxybutyrate (BOHB) levels were recorded daily using a mobile interface. Hemoglobin A_1c_ (HbA_1c_) and related biomarkers of T2D were evaluated at baseline and 10-week follow-up.

**Results:**

Baseline HbA_1c_ level was 7.6% (SD 1.5%) and only 52/262 (19.8%) participants had an HbA_1c_ level of <6.5%. After 10 weeks, HbA_1c_ level was reduced by 1.0% (SD 1.1%; 95% CI 0.9% to 1.1%, *P*<.001), and the percentage of individuals with an HbA_1c_ level of <6.5% increased to 56.1% (147/262). The majority of participants (234/262, 89.3%) were taking at least one diabetes medication at baseline. By 10 weeks, 133/234 (56.8%) individuals had one or more diabetes medications reduced or eliminated. At follow-up, 47.7% of participants (125/262) achieved an HbA_1c_ level of <6.5% while taking metformin only (n=86) or no diabetes medications (n=39). Mean body mass reduction was 7.2% (SD 3.7%; 95% CI 5.8% to 7.7%, *P*<.001) from baseline (117, SD 26, kg). Mean BOHB over 10 weeks was 0.6 (SD 0.6) mmol·L^−1^ indicating consistent carbohydrate restriction. Post hoc comparison of the remote versus on-site means of education revealed no effect of delivery method on change in HbA_1c_ (*F*_1,260_=1.503, *P*=.22).

**Conclusions:**

These initial results indicate that an individualized program delivered and supported remotely that incorporates nutritional ketosis can be highly effective in improving glycemic control and weight loss in adults with T2D while significantly decreasing medication use.

## Introduction

Type 2 diabetes is generally regarded as a chronic, progressive disease that can be slowed by the vigorous use of lifestyle changes and medications but eventually results in vascular damage and end-organ failure [1,2]. Current medical treatment interventions result in virtually no disease remission, as seen in a study within the Kaiser health care population where the spontaneous remission rate is 0.5% [3]. As the disease progresses, it has been shown that glucose-lowering medication use, health care costs, and complications all rise. At 9 years, less than 25% of patients are able to control their blood glucose level with only one medication [4], and 10-15 years after the diagnosis of type 2 diabetes, more than 50% of patients will require insulin [5].

Despite the overall paucity of type 2 diabetes remission data, there exist three notable treatment exceptions. Bariatric surgery, such as gastric bypass, is effective at reversing type 2 diabetes, with 40%-60% of surgical patients demonstrating remission 1 year after the surgery. The most comprehensive study of surgical intervention to prevent or reverse type 2 diabetes is the Swedish Obese Subjects Trial [6], demonstrating an 8-fold reduction in the incidence of the disease at 2 years. However, further out into the postoperative experience, many of these patients regain weight and relapse into diabetes, and they are at risk of developing nutritional deficiencies as well [7].

There have been many reports of short-term improvement in glycemic control with very low-calorie diets (VLCDs) consisting of either common foods or chemically defined formulas, ranging in energy from 400-800 kcal·day^−1^. Bistrian et al [8] administered a common-food 600-800 kcal·day^−1^ VLCD to 7 insulin-using subjects with type 2 diabetes for inpatient and outpatient durations of 2-12 months. All 7 subjects achieved rapid improvement in glycemic control despite the cessation of insulin therapy, and 6 of 7 subjects experienced substantial weight loss. Bauman et al [9] hospitalized 64 patients with type 2 diabetes, including 42 patients taking insulin, and administered a VLCD for a mean of 23 days. After 19 months, 10 patients remained in remission. Wing et al [10] randomized 93 obese individuals with type 2 diabetes to either a low-calorie diet or an intermittent formula VLCD for 1 year. The VLCD group achieved greater initial weight loss and greater hemoglobin A_1c_ (HbA_1c_) reductions, but these differences between the 2 diet arms were not sustained over the duration of the study. In a recent study by Steven et al [11], 13 of 30 individuals with type 2 diabetes but not using insulin achieved normal blood glucose values after 8 months of lifestyle intervention. In this case, a chemically defined, liquid, low-carbohydrate VLCD was prescribed for 8 weeks, followed by 6 months of an unspecified energy maintenance diet.

These 4 studies [8-11] used VLCDs to control blood glucose level while stopping or reducing diabetes medications. The limitation of using a VLCD to manage a chronic disease is that this type of diet is necessarily temporary, given that it provides less than 800 kcal·day^−1^ and thus is unsustainable in the long term.

Alternatively, nutritional ketosis, defined as a dietary regimen resulting in serum beta-hydroxybutyrate (BOHB) levels between 0.5 and 3.0 mmol·L^−1^ [12], may yield similar or better results over longer periods of time by not explicitly prescribing caloric restriction. Nutritional ketosis is often achieved by reduced carbohydrate, moderate protein, and increased fat intake. In this setting, moderately reduced energy intake may occur in association with the proportionately high fat intake, reduced circulating insulin due to reduced carbohydrate consumption, and potential metabolic benefits of mild ketonemia. For example, Boden et al [13] reported that in patients with type 2 diabetes fed a ketogenic diet to satiety improved insulin sensitivity by 75% within 2 weeks. When given free access to a ketogenic buffet, daily energy intake dropped by about one-third, resulting in a total weight loss of 2 kg over 2 weeks. The authors concluded that this modest weight loss in and of itself could not explain the improved insulin sensitivity.

There have been a number of studies using low-carbohydrate, high-fat dietary strategies in the management of type 2 diabetes [14-20], but these group sizes have been small and often excluded subjects taking insulin. In addition, the dietary interventions used in these studies frequently were not sufficiently low in carbohydrate or protein to induce sustained nutritional ketosis. However, multiple studies of ketogenic diets prescribed without energy restriction have demonstrated both tolerability and effectiveness of this dietary approach to improve a broad range of cardiometabolic markers in prediabetic and dyslipidemic outpatients [21-23]. And finally, recent studies have identified BOHB in the nutritional ketosis range as a potent epigenetic signal that decreases oxidative stress [24], hepatic glucose output [25], and insulin resistance [26].

We therefore hypothesized that a comprehensive program with individualized nutritional recommendations that supports participants in achieving sustained nutritional ketosis while eating to satiety may have unique benefits in the management of type 2 diabetes. Specifically, this study was designed to assess the practical utility of an intensive digital intervention supported by medical management, continuous digital health coaching, nutrition education, behavioral support, biometric feedback, and peer support via an online community. We refer to this technology-enabled medical service as the Virta Clinic.

## Methods

### Subjects

Adults with type 2 diabetes between the ages of 21 and 65 years were recruited via clinical referrals, media advertising, and word of mouth in the greater Lafayette, Indiana, region. Exclusion criteria included advanced renal, cardiac, and hepatic dysfunction, history of ketoacidosis, dietary fat intolerance, or pregnancy or planned pregnancy.

### The Virta Clinic

Virta utilizes a technology-enabled, full-service clinic model for metabolic recovery from type 2 diabetes including medical management by physicians, health coaching, nutrition and behavior change education, biometric feedback, and peer support. Physicians and health coaches were trained in the basic principles of achieving and maintaining nutritional ketosis based on previous published works [21,22,27]. In this study, educational content was delivered via either on-site weekly 90-minute group-based classes or Web-based recorded educational content, and participants self-selected their preferred mode of content delivery. The same educational content was provided by each delivery method. Educational content included discussion of the pathophysiology of diabetes, practical management of carbohydrate restriction while consuming protein in moderation and increasing fat intake, the utilization of ketones as a biofeedback mechanism, and appropriate utilization of behavior change techniques. No modifications to participants’ physical activity were encouraged in the first 10 weeks of the intervention.

Remote support was provided to each subject through tracking of daily biometrics, the assignment of a personal health coach available daily via one-on-one texting for advice and problem solving, support via an online community of his or her peers, and physician supervision. Subjects were instructed to monitor and report glucose level via the Web to the care team 1-3 times per day, and a physician made medication changes as appropriate. Additionally, the medication status of each participant was reviewed by the care team and the principal investigator weekly.

### Nutritional Ketosis

The Virta Clinic includes individualized nutritional recommendations to sustain nutritional ketosis by titrating carbohydrate and protein intake to the patient’s individual tolerance [27]. With the insulin resistance characteristic of type 2 diabetes, subjects typically require total dietary carbohydrates to be restricted to <30 g·day^−1^. Daily protein intake was targeted to a level of 1.5 g·kg^−1^ of reference (ie, medium-frame “ideal”) body weight and participants were coached to incorporate dietary fats to satiety. Other aspects of the diet were individually prescribed to ensure safety, effectiveness, and satisfaction, including consumption of 3-5 servings of nonstarchy vegetables and adequate mineral and fluid intake for the ketogenic state. BOHB was monitored routinely via finger-stick blood monitoring using a handheld device (Abbott Precision Xtra Blood Glucose and Ketone Monitoring System, Alameda, CA, USA) and participants were encouraged to obtain BOHB readings ≥0.5 mmol·L^−1^.

### Outcome Measures and Testing Procedures

Type 2 diabetes status was determined by HbA_1c_ level at baseline and again at 10-11 weeks into the program. A value of 6.5% or greater, or HbA_1c_ level <6.5% but taking at least one hypoglycemic medication, was considered indicative of type 2 diabetes. Secondary outcome measures included assessment of (1) body weight determined daily on a cellular-connected scale (BodyTrace BT003 cellular-connected scale, New York, New York, USA); (2) medication use for control of diabetes; and (3) blood pressure obtained in the seated position. Fasting blood was analyzed for total cholesterol, low-density lipoprotein cholesterol, high-density lipoprotein cholesterol, triglycerides, C-reactive protein, total white blood cell count, and kidney and liver functions. All laboratory test results were analyzed by standard procedures. Hunger was assessed using a 4-point Likert scale from 1 (no) to 4 (always), representing the participant’s subjective level of hunger over the previous 24-hours.

### Statistical Analysis

Descriptive statistics were calculated for each variable as mean (SD). Baseline and 10- to 11-week follow-up measures were compared with paired-sample *t* tests to evaluate for significant differences in primary (HbA_1c_ level) and secondary outcome variables over time, following implementation of carbohydrate restriction per the Virta Clinic. Statistical significance was set a priori at *P*<.05; for secondary outcome variables, we applied a Bonferroni adjustment for multiple comparisons, setting *P*<.003 as the level of significance for those outcome measures. McNemar test with continuity correction and Bonferroni adjustment for multiple comparisons was utilized to assess for a difference in the proportion of participants who were prescribed each of the 7 medication classes at baseline compared with follow-up, setting *P*<.007 as the level of significance. We utilized an intention-to-treat analysis with the last observation carried forward for analyses of all participants; separate subanalyses were performed for participants who completed follow-up testing (completers). Given that 2 different modes were utilized for delivery of educational content, we performed a post hoc analysis on the primary outcome measure to determine if differences existed between groups.

### Institutional Review Board Approval

The protocol was reviewed and approved by the Institutional Review Board at Franciscan Health Lafayette East, Lafayette, Indiana. Subjects were informed of the purpose and possible risks of the investigation before signing an informed consent document approved by the institutional review board.

## Results

### Characteristics of Subjects

A total of 262 subjects with diagnosis of type 2 diabetes were enrolled in this study. The mean age was 54 (SD 8) years and 66.8% (175/262) were female. Additional baseline data are provided in Table 1.

**Table 1:** Participant characteristics at baseline and follow-up.

Characteristics		n^a^	Baseline	Follow-up	Mean difference		*t*_n-1_			*P*^b^	
			Mean (SD)	Mean (SD)	Mean (SD)	95% CI					
**Hemoglobin A**_1c_**(%)**											
	All	262	7.6 (1.5)	6.6 (1.1)	−1.0 (1.1)	−1.1 to −0.9	14.9			<.001	
	Completers	238	7.6 (1.5)	6.5 (1.0)	−1.1 (1.1)	−1.2 to −1.0	15.6			<.001	
**Fasting glucose (mg·dL^−1^)**											
	All	259	162 (61)	131 (37)	−30 (56)	−37 to −25	8.68			<.001	
	Completers	236	163 (62)	129 (34)	−33 (58)	−41 to −26	8.8			<.001	
**Body mass index (kg·m^−2^)**											
	All	262	40.8 (8.9)	37.9 (8.5)	−2.9 (1.2)	−3.1 to −2.7	30			<.001	
	Completers	238	40.7 (8.5)	37.7 (8.0)	−3.1 (1.5)	−3.3 to −2.9	31.3			<.001	
**Weight (kg)**											
	All	262	117 (26.3)	109 (24.9)	−8 (4.6)	−9 to −8	29.1			<.001	
	Completers	238	117 (25.7)	109 (24.3)	−9 (4.5)	−9 to −8	30.7			<.001	
**Systolic blood pressure (mm Hg)**											
	All	260	132 (16)	126 (15)	−6 (19)	−8 to −4	5.29			<.001	
	Completers	236	132 (17)	125 (15)	−7 (20)	−9 to −4	5.32			<.001	
**Diastolic blood pressure (mm Hg)**											
	All	260	82 (10)	78 (10)	−4 (12)	−5 to −2	5.22			<.001	
	Completers	236	82 (10)	78 (9)	−4 (12)	−6 to −3	5.25			<.001	
**Total cholesterol (mg·dL^−1^)**											
	All	262	177 (41)	172 (41)	−5 (31)	−9 to −1	2.64			.009	
	Completers	238	177 (41)	172 (41)	−6 (33)	−10 to −1	2.64			.009	
**LDL-C^c^(calculated; mg·dL^−1^)**											
	All	245	97 (33)	99 (36)	2 (25)	−2 to 5	0.987			.32	
	Completers	223	98 (34)	99 (37)	2 (27)	−2 to 5	0.987			.32	
**HDL-C^d^(mg·dL^−1^)**											
	All	262	44 (13)	44 (13)	0.5 (8)	−0.5 to 1	0.966			.33	
	Completers	238	44 (14)	45 (13)	0.5 (8)	−0.5 to 1.5	0.966			.33	
**Triglycerides (mg·dL^−1^)**											
	All	262	185 (127)	147 (87)	−37 (107)	−50 to −24	5.61			<.001	
	Completers	238	185 (129)	145 (84)	−41 (112)	−55 to −27	5.64			<.001	
**Serum creatinine (mg·dL^−1^)**											
	All	259	0.88 (0.24)	0.85 (0.22)	−0.03 (0.12)	−0.04 to −0.01	3.61			<.001	
	Completers	236	0.88 (0.24)	0.85 (0.22)	−0.03 (0.13)	−0.05 to −0.01	3.61			<.001	
**ALT^e^(units·L^−1^)**											
	All	259	31 (23)	26 (16)	−4 (19)	−7 to −2	3.82			<.001	
	Completers	236	31 (24)	26 (16)	−5 (20)	−7 to −2	3.83			<.001	
**AST^f^(units·L^−1^)**											
	All	259	24 (15)	21 (9)	−3 (13)	−4 to −1	3.31			<.001	
	Completers	236	24 (16)	21 (9)	−3 (14)	−5 to −1	3.31			<.001	
**Alkaline phosphatase (units·L^−1^)**											
	All	259	74 (22)	68 (20)	−6 (11)	−8 to −5	9.78			<.001	
	Completers	236	75 (22)	67 (20)	−8 (11)	−9 to −6	9.96			<.001	
**C-reactive protein (mg·L^−1^)**											
	All	247	8.1 (8.2)	9.2 (11.5)	1.2 (7.5)	−0.2 to 2.1	2.45			.01	
	Completers	225	8.2 (8.1)	9.6 (12.1)	1.4 (8.1)	−0.3 to 2.1	2.6			.01	
**Total WBC^g^(x10^9^·L^−1^)**											
	All	236	7.2 (1.9)	6.7 (1.9)	−0.5 (1.3)	−0.6 to −0.3	5.37			<.001	
	Completers	234	7.2 (1.8)	6.7 (1.9)	−0.5 (1.3)	−0.6 to −0.3	5.36			<.001	

^a^Reductions in the number of participants (n) are due to missed laboratory orders, except in the case of LDL-C, where LDL-C was incalculable.

^b^We set *P*<.003 as the level of significance for multiple comparisons.

^c^LDL-C: low-density lipoprotein cholesterol.

^d^HDL-C: high-density lipoprotein cholesterol.

^e^ALT: alanine aminotransferase.

^f^AST: aspartate aminotransferase.

^g^WBC: white blood cell.

### Retention

At 11 weeks, 21 of the 262 subjects had dropped out and 3 had not obtained the follow-up laboratory test results, yielding 238 or 90.8% retention for this phase of the study. Among the noncompleters, the most common reasons to leave the study were as follows: removed for noncompliance (n=6), unrelated health issue took priority (n=3), family illness or other issues (n=3), cost of medical appointments (n=2), and undisclosed personal choice (n=2). The age and sex distributions did not differ between noncompleters and completers.

### Program Adherence

**Figure 1:**
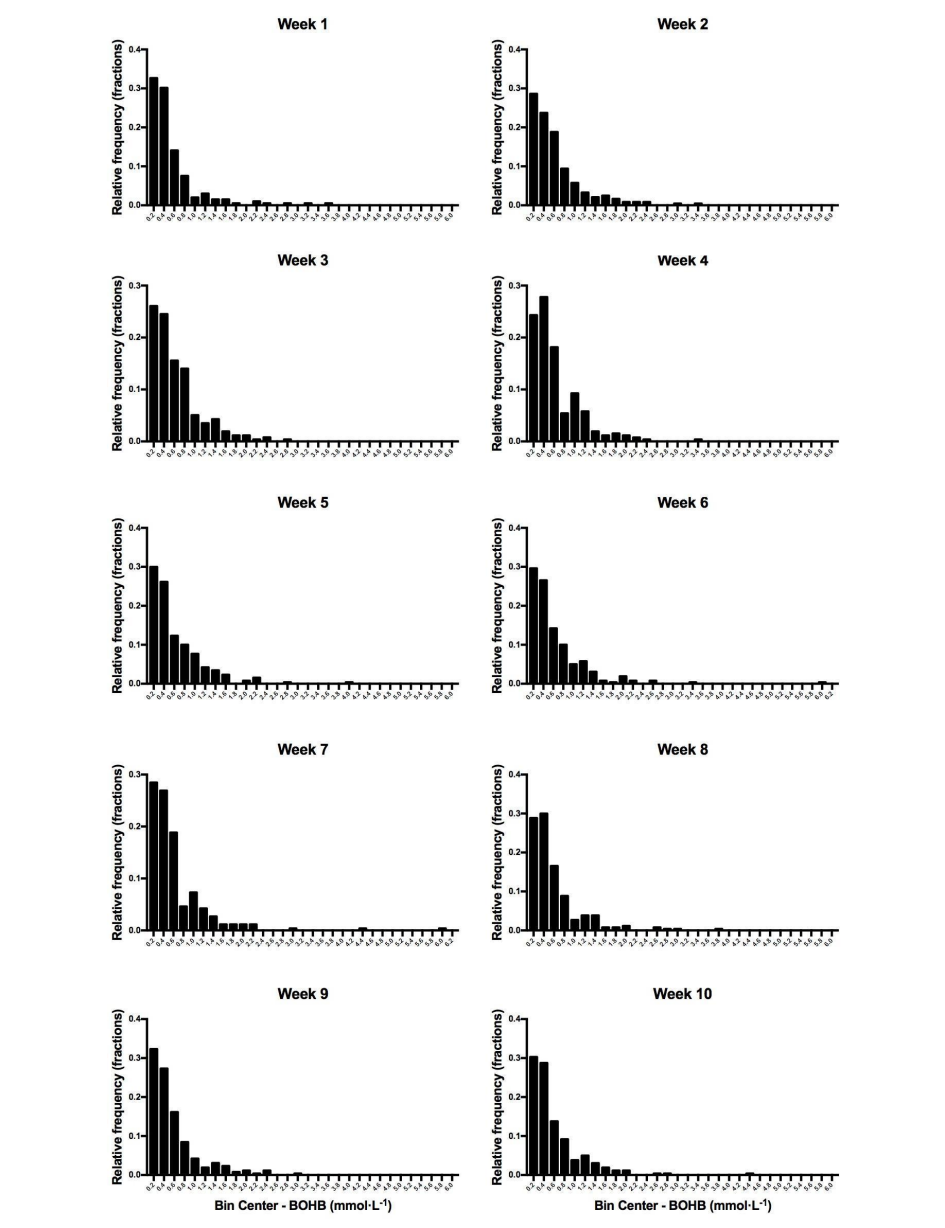
Relative frequency distribution of participant weekly average beta-hydroxybutyrate (BOHB) concentrations. An observed weekly average BOHB concentration on the border of 2 bins is placed in the bin holding the larger values. Evidence of carbohydrate restriction exhibited by elevated ketones was present in the first week in the majority of subjects and maintained for the duration of the study. All reported BOHB concentrations greater than 3.0 were in participants taking a sodium-glucose cotransporter-2 inhibitor, except for one (4.4 mmol•L−1) in which we suspect elevated BOHB due to increased exercise and another (6.0 mmol•L−1) in which we suspect participant data entry error. Excluding this 1 value, average BOHB concentrations for this participant ranged from 0.4 to 1.4 mmol•L^−1^.

Daily BOHB level averaged over 10 weeks of the program was 0.6 (SD 0.6) mmol·L^−1^ (see Figure 1). This range is indicative of a modest state of nutritional ketosis in most of the subjects, with highest value similar to levels observed during fasting. There were no cases of diabetic ketoacidosis (ie, hyperglycemia concurrent with serum BOHB level >6 mmol·L^−1^).

### Hemoglobin A1c

**Figure 2:**
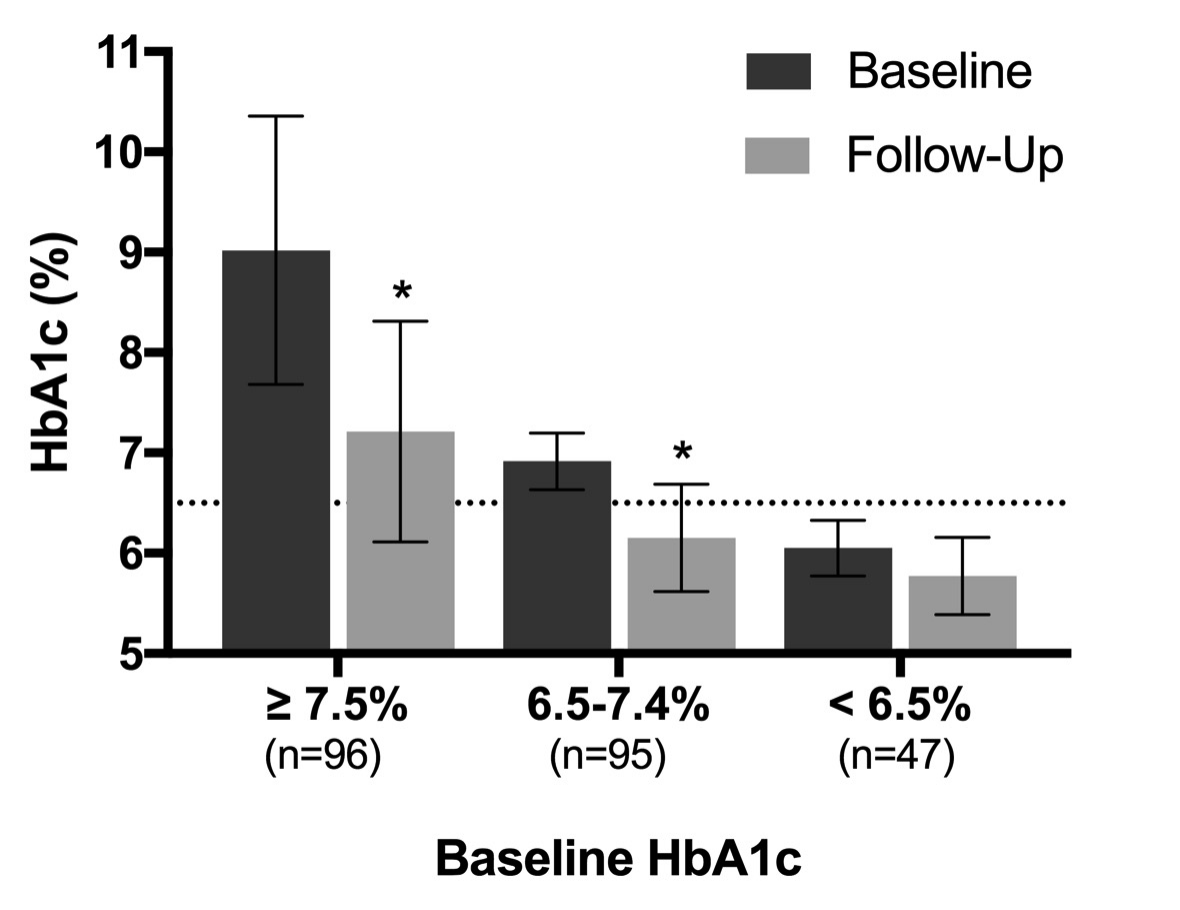
Hemoglobin A1c (HbA1c) changes by baseline level. Error bars represent SD; the dotted line represents the threshold for diagnosis of type 2 diabetes. Significant reductions in HbA1c level from baseline to follow-up were observed in subjects whose baseline HbA1c level was ≥7.5% (mean 9.0%, SD 1.3% to 7.2%, SD 1.1%, *P*<.001) and between 6.5% and 7.4% (mean 6.9%, SD 0.3% to 6.2%, SD 0.5%, *P*<.001). For those whose baseline HbA1c level was <6.5%, HbA1c level was improved but not significantly after correcting for multiple comparisons (mean 6.1%, SD 0.3% to 5.8%, SD 0.4%, *P*=.03). *Represents significant difference from baseline.

Baseline HbA_1c_ level was 7.6% (SD 1.5%) and 210/262 (80.2%) participants had an HbA_1c_ level of ≥6.5%. After 10 weeks, HbA_1c_ level was reduced by 1.0% (SD 1.1%; 95% CI 0.9% to 1.1%, *P*<.001), and 56.1% (147/262) achieved an HbA_1c_ level of <6.5%. HbA_1c_ level for the 238 completers was similarly reduced from 7.6% (SD 1.5%) at baseline to 6.5% (SD 1.0%; 95% CI of mean difference −1.2% to −1.0%, *P*<.001) at 10-11 weeks into the Virta Clinic program. The varying responses of HbA_1c_ based upon starting level are shown in Figure 2. Of the 147 participants who achieved an HbA_1c_ level of less than 6.5%, 143 (97.3%) reached this goal without an increase in the number or dosage of diabetes medications. At follow-up, 47.7% of participants (125/262) achieved an HbA_1c_ level of less than 6.5% while taking metformin only (n=86) or no diabetes medications (n=39).

Post hoc analysis of method of educational content delivery revealed there was no significant interaction between delivery method and time for HbA_1c_ (*F*_1,260_=0.18, *P*=.67), nor was there an effect of delivery method (*F*_1,260_=1.503, *P*=.22). Baseline HbA_1c_ level was similar (on-site: mean 7.7%, SD 1.6%, digital: mean 7.5%, SD 1.4%; mean difference = 0.2%, 95% CI of mean difference: −0.2% to 0.5%; *t*_520_=0.94, *P*=.69), and HbA_1c_ reductions of 1.0% (SD 1.1%) and 1.0% (SD 1.0%) for the on-site and digital content delivery methods, respectively, were achieved with no difference between delivery methods at follow-up (mean difference = 0.2%, 95% CI of mean difference: −0.2% to 0.6%; *t*_520_=1.29, *P*=.39).

### Hypoglycemic Medications

**Figure 3:**
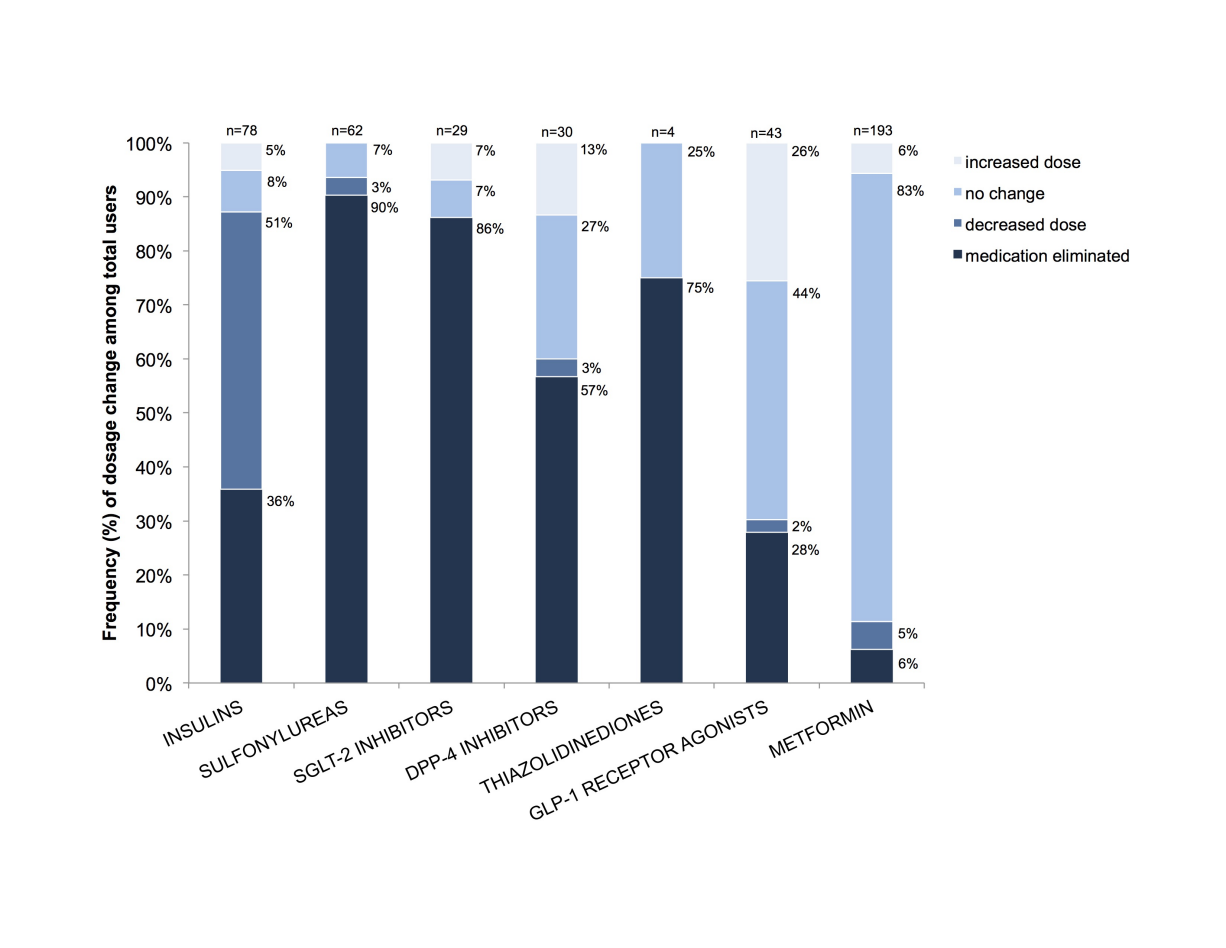
Frequency of medication dose changes by drug class. Bars represent total users of each drug with the type of dose change (increase, no change, decrease, or elimination) stacked within the bar and the relative frequency noted next to each section. The total number of users is noted at the top of each bar. The proportion of participants who were prescribed the drug was significantly different between baseline and follow-up for insulin (χ2_1_=21.4, *P*<.001), sulfonylureas (χ2_1_=54.0, *P*<.001), and sodium-glucose cotranporter-2 (SGLT-2) inhibitors (χ2_1_=17.9, *P*<.001) but not for dipeptidyl peptidase-4 (DPP-4) inhibitors (χ2_1_=6.9, *P*=.009), thiazolidinediones (χ2_1_=1.3, *P*=.25), glucagon-like peptide-1 (GLP-1) receptor agonists (χ2_1_=0.5, *P*=.50), or metformin (χ2_1_=0.8, *P*=.36).

The majority of participants (234/262, 89.3%) were taking at least one diabetes medication at baseline. Both the number and dosage of most diabetes medications were reduced substantially in the first 10-11 weeks of the Virta Clinic program (Table 2, Figure 3). As shown in Table 2, of the initial 262 subjects, 112 (42.7%) experienced a decrease in their medications with another 21 (8.0%) having their medications eliminated. Only 13 (5.0%) of the 262 subjects were prescribed a new class or increased dose of medication. Of the 262 participants, 88 (33.6%) had no change in their medications and 28 (10.7%) were taking no hypoglycemic medications at entry into the study or at follow-up.

**Table 2:** Change in prescription of medication class or dose between baseline and follow-up.

Change in medication prescription or dose between baseline and follow-up	n	HbA_1c_^a^ <6.5% at follow-up, n (%)	Baseline HbA_1c_ (%), mean (SD)	Follow-up HbA_1c_ (%), mean (SD)
Increase	13	4 (31)	8.5 (2.0)	7.4 (1.4)
No change	88	57 (65)	7.2 (1.2)	6.5 (1.0)
Decrease	112	47 (42)	8 (1.6)	6.8 (1.1)
Complete elimination of medications	21	17 (81)	6.7 (0.9)	6.1 (0.5)
No medications prescribed	28	22 (79)	7.3 (1.3)	6.3 (1.1)

^a^HbA_1c_: hemoglobin A_1c_.

Figure 3 shows the changes in the 7 common classes of hypoglycemic medication prescribed to the subjects in this study. For sulfonylureas, sodium-glucose cotransporter-2 inhibitors, and thiazolidinediones, the vast majority of subjects discontinued these medications (90.3%, 86.2%, and 75.0%, respectively). To a lesser degree, this was also the case for dipeptidyl peptidase-4 inhibitors (56.7%), insulin (35.9%), and glucagon-like peptide-1 receptor agonists (27.9%). The exception to this trend was metformin. The proportion of participants who were prescribed insulin, sulfonylureas, and sodium-glucose cotransporter-2 inhibitors was significantly different at follow-up compared with baseline (all *P*<.007, see Figure 3). Given the reduced risk for hypoglycemia with the glucagon-like peptide-1 receptor agonists relative to insulin and sulfonylureas, the former was added in some cases in order to withdraw the latter two. In the case of metformin, given its modest but significant efficacy in the prevention of diabetes, its continued use in this cohort was encouraged (ie, 186 users at baseline and 181 at follow-up).

**Figure 4:**
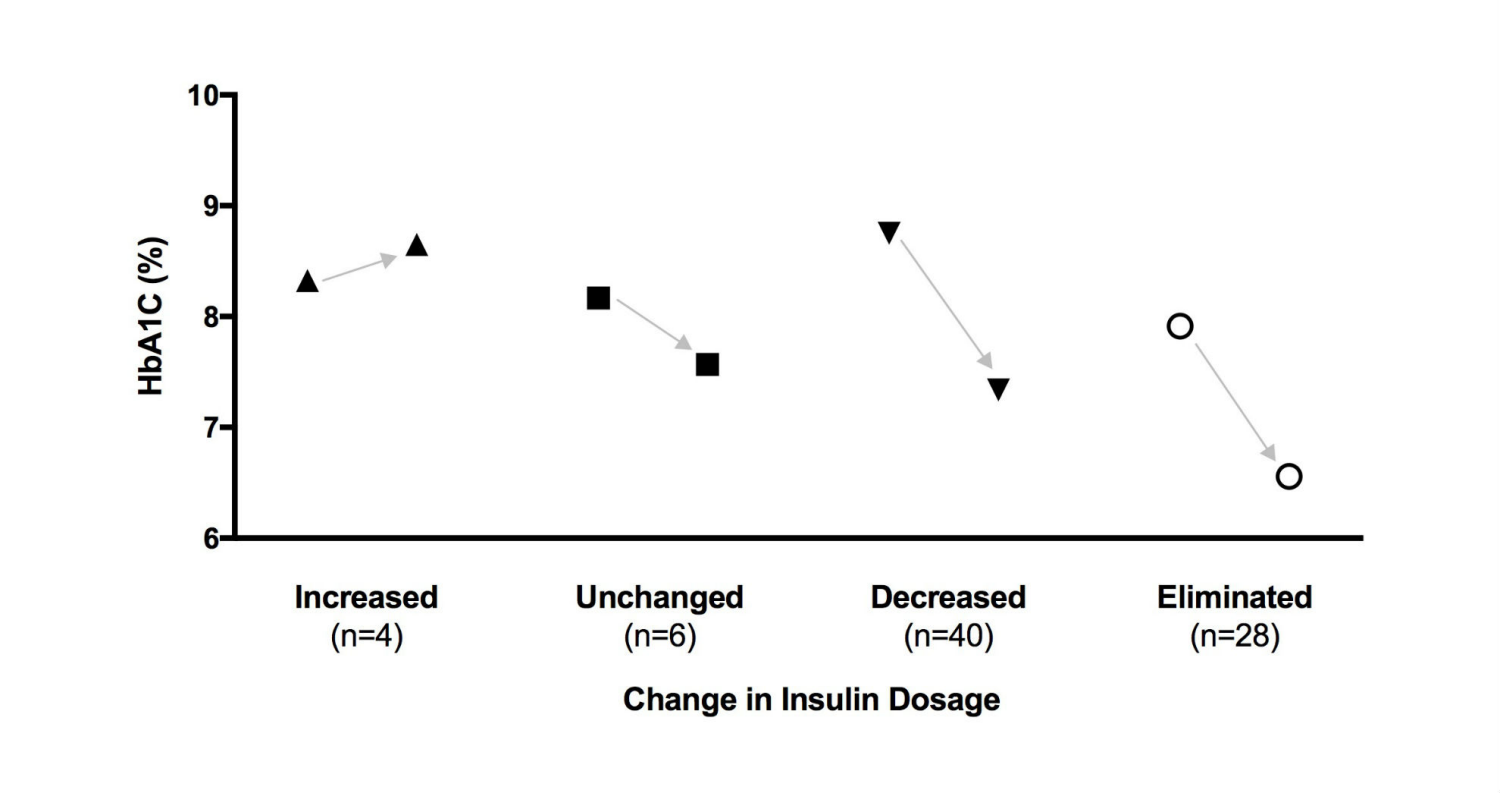
Hemoglobin A1c (HbA1c) level at baseline and 10-11 weeks per change in insulin dosage. Insulin users who were able to eliminate or reduce their use of the drug also significantly reduced their HbA1c level (7.9%, SD 1.5%, to 6.6%, SD 0.9%, P<.001 and 8.8%, SD 1.8%, to 7.4%, SD 1.2%, P<.001, respectively). Six users with no change in insulin dose achieved a reduction in HbA1c level, although it was not statistically significant (8.2%, SD 1.8%, to 7.6%, SD 1.2%, P=.25). Despite an increased insulin dosage in 4 users, HbA1c level increased but the difference was not significant (8.3%, SD 0.4%, to 8.7%, SD 0.8%, P=.61).

Figure 4 shows changes in HbA_1c_ level over 10-11 weeks in subjects whose insulin dosage was increased, unchanged, reduced, or eliminated. Only 5% (4 of 78 initial users) had their dosage increased in order to manage their initial HbA_1c_ value of 8.3% (SD 0.4%). For the other 74 subjects who entered the study while taking insulin, the HbA_1c_ values declined significantly despite the same, reduced, or eliminated insulin dosages.

### Body Weight

**Figure 5:**
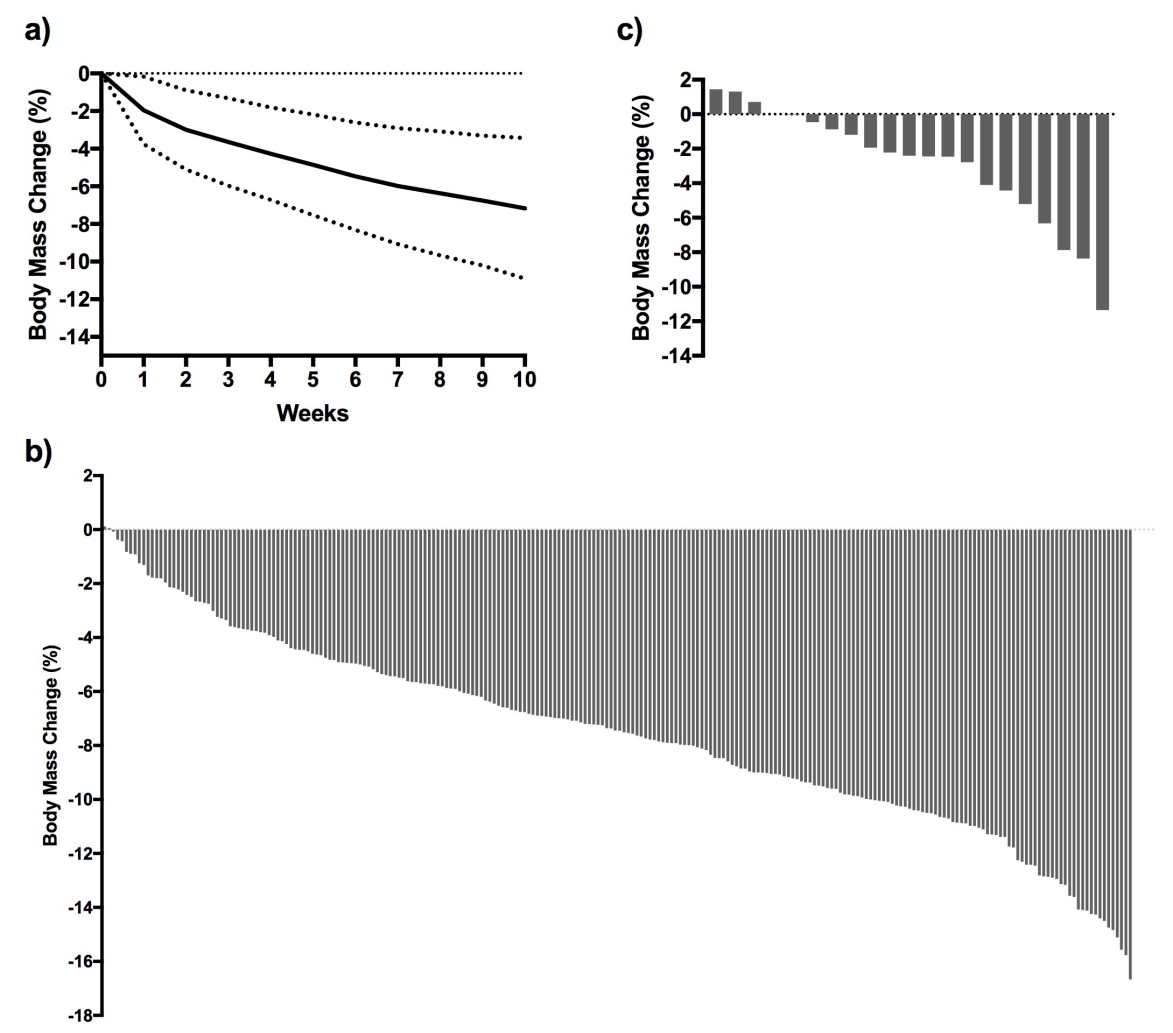
Participant weight loss over 10 weeks. Part “a”—weight change over 10 weeks for all participants. Solid line represents the mean; dotted lines represent one standard deviation from the mean. Part “b”—individual body weight changes as percentage of starting body weight at 10 weeks for completers (n=238). Part “c”—individual body weight changes as percentage of starting body weight for each noncompleter at the time of removal from study. For the 21 dropouts, time to drop out was 6 (SD 3) weeks (n=3 participants are still enrolled in the study but did not complete 10-week follow-up testing).

Weight and body mass index (BMI) changes from baseline to 10 weeks are presented in Table 1, and the mean weight change (as percentage of starting weight) over time is shown in Figure 5 (part “a”). Figure 5 (parts “b” and “c”) also shows individual subjects’ weight change over 10 weeks for completers and at the time of dropout for noncompleters. Mean weight loss at 10 weeks for completers was 7.2% (SD 3.7%) of initial body weight. Only 5 out of 262 subjects (2 completers, 3 noncompleters) registered a weight gain, and 75% of completers lost 5% or more of their initial body weight in this time period.

### Laboratory Test Results and Measures

Consistent with the HbA_1c_ changes, the fasting glucose level (Table 1) declined markedly despite reduced hypoglycemic medication usage. There were no significant changes in total, low-density lipoprotein, or high-density lipoprotein cholesterol levels, nor were any changes made to statin prescriptions during this time. Triglycerides were significantly reduced by 20%. There were modest but significant reductions in both systolic and diastolic blood pressure. Although not elevated at baseline, the mean serum creatinine, alanine aminotransferase, aspartate aminotransferase, and alkaline phosphatase levels were all significantly reduced at 10-11 weeks. Biomarkers of inflammation were mixed. While C-reactive protein was unchanged, total white blood cell count decreased significantly after 10 weeks of the ketogenic diet.

Baseline hunger on a scale from 1 (no) to 4 (always) was 1.6 (SD 0.6). At 10 weeks, subjective hunger was 1.3 (SD 0.4; 95% CI of mean difference: −0.4 to −0.2, *t*_134_=5.58, *P*<.001). Furthermore, 46/135 (34.1%) subjects at baseline reported no hunger, increasing to 78/135 (57.8%) at 10 weeks.

### Side Effects

One subject withdrew from the study in the first 70 days because of a dietary side effect (diarrhea due to fat intolerance). There were no serious adverse events in this time period and, specifically, no serious symptomatic hypoglycemic events requiring medical intervention.

## Discussion

Although the American Diabetes Association has recently relaxed its advocacy for severe dietary fat restriction, the current paradigm for the management of type 2 diabetes is to prescribe a diet containing about 40% of energy from carbohydrates (eg, a Mediterranean diet) and then adjust medications as necessary to maintain glycemic control [28]. The Virta Clinic manages type 2 diabetes from the perspective that it is a disease of carbohydrate intolerance. Given that this investigation is a nonrandomized demonstration study without measurement against standard of care, no statistical comparisons are made. However, these data demonstrate that when participants were supported through a novel, individualized program including instruction for limiting dietary carbohydrates to <30 g·day^−1^, medications could be substantially reduced or eliminated in most subjects, overall glycemic control was improved, and clinically relevant weight loss (5% or greater) was achieved in a majority of participants.

Other group-based and digitally delivered programs have demonstrated improvements in HbA_1c_ level with modest or no reduction in weight and often without a reduction in medication. A recent in-person group-based intervention for weight loss in adults with type 2 diabetes reduced HbA_1c_ level by 0.7% and weight by 3.3% after 12-13 weeks [29], while our investigation reduced HbA_1c_ level by 1.0% and weight by 7.2% in 10-11 weeks. Digitally delivered programs have elicited a range of improvements in HbA_1c_ (from none to significant) [30]; however, these results were often achieved by increased medication use due to improved adherence and without a reduction in weight [31]. This study demonstrated that these results (reduced HbA_1c_ level, weight, and medication use) can be achieved concurrently. Specifically, 147 (56.1%) of the initial 262 subjects in this initial study of the Virta Clinic registered HbA_1c_ values <6.5% at 10- to 11-week follow-up. Of these, 39 participants were able to achieve these results without taking any diabetes medication and 86 participants were able to achieve these results taking only metformin. Considering the equilibration time for HbA_1c_ is approximately 120 days, the significant decrease after 70-77 days reported here is a conservative estimate of the true improvement in glucose metabolism.

Achieving an HbA_1c_ value under 6.5% is considered “tight control” for type 2 diabetes. There are two commonly reported side effects of tight control—weight gain [32,33] and symptomatic hypoglycemia [28,33,34]. Paradoxically, in this study, we observed very consistent weight loss while observing no severe symptomatic hypoglycemic events. In addition to the very close mobile communication between the participant, coach, and physician in the Virta Clinic, this absence of severe hypoglycemic episodes despite very tight glucose control may be due to the protection of central nervous system function by circulating levels of BOHB. Two studies of starvation-adapted humans have demonstrated full preservation of central nervous system function despite profound hypoglycemia induced by exogenous insulin administration [35,36].

As it pertains to weight loss, it is all the more interesting that the Virta Clinic instructs its participants to strictly limit carbohydrates and eat protein in moderation but to eat fat to satiety. In daily Web-based questionnaires, patients reported reduced hunger once adapted to the ketogenic diet. This subjective decrease in hunger, albeit modest in magnitude, may have allowed the majority of subjects to experience significant weight loss. This concurrent combination of weight loss and reduced hunger is particularly interesting given that significant weight loss by caloric restriction typically increases hunger [37]. However, in light of the recent reports of epigenetic effects of BOHB reducing oxidative stress [24,38] and improving insulin sensitivity [26], it is possible that these paradoxical results can be ascribed to a combination of the metabolic and epigenetic effects of mild nutritional ketosis.

Although we have not calculated the economic implications of improved glycemic control with reduced medications, the removal of diabetes medications combined with clinically significant weight loss [39] has been shown to generate health care cost savings. The timing of these cost savings is immediate in the case of the medication reductions and could accrue over time because of the effect of lowering BMI. As for the HbA_1c_ reduction observed in this study, when changes of this magnitude are attained with intensive medication use, this tends to increase both drug costs and adverse events [40]. However, given that a 0.5% reduction in HbA_1c_ level was associated with a 17% reduction in diabetic vascular complications following aggressive medication management [2], our 1.0% HbA_1c_ level reduction with less medication has the potential to yield even greater savings in the cost of complications over time.

In conclusion, we demonstrated for the first time that biomarkers of type 2 diabetes can be reversed in a substantial fraction of participants using a comprehensive digitally delivered intervention, including medical management by physicians, health coaching, nutrition education emphasizing individualized carbohydrate intake to induce nutritional ketosis, behavioral support, biometric feedback, and peer support. In contrast to current intensive pharmaceutical management strategies, the positive results were achieved with less use of medication and substantial weight loss. The brief duration of this initial study cannot predict the long-term outcomes or sustainability of the nutrition recommendations used by the Virta Clinic. Early results demonstrate markedly improved glycemic control with less medication and modest changes in blood pressure, total white blood cell count, and liver and kidney functions. Ongoing work will evaluate the efficacy and sustainability of this intervention over 2 years.

## Abbreviations

BMI: body mass index
BOHB: beta-hydroxybutyrate
HbA1c: hemoglobin A1c
VLCD: very low-calorie diet
